# Implementation of antibiotic stewardship in different settings - results of an international survey

**DOI:** 10.1186/s13756-019-0493-7

**Published:** 2019-02-12

**Authors:** E. Charani, Enrique Castro-Sanchéz, S. Bradley, D. Nathwani, Alison H. Holmes, P. Davey

**Affiliations:** 1 NIHR Health Protection Research Unit in Healthcare Associated Infections and Antimicrobial Resistance, Hammersmith Campus, Imperial College London, W12 ONN London, England; 2 British Society for Antimicrobial Chemotherapy, 53 Regent Place, Birmingham, England; 3 University of Dundee, Medical School, Dundee, Scotland

**Keywords:** Antibiotic stewardship, Education, Culture, Antibiotic prescribing

## Abstract

**Background:**

Antibiotic stewardship interventions are being implemented across different healthcare settings. We report the findings of a global survey of healthcare professionals on the implementation of antibiotic stewardship programmes.

**Methods:**

Learners of a Massive Online Open Course (MOOC) on antibiotic stewardship were invited to complete an online survey on the core available organisational resources for stewardship. The categorical variables were analysed using chi-squared test, and Likert questions were analysed using an ordinal regression model. The *p*-values were considered as two-tailed. Significance was set at p-value of < 0.05.

**Results:**

The response rate was 55% (505/920), from 53 countries. The responders were 36% (182) doctors, 26% (130) pharmacists, 18% (89) nurses and 20% (104) other (researchers, students and members of the public). Post-graduate training in infection management and stewardship was reported by 56% of doctors compared with 43% (OR 0.59, 95%CI 0.35–1.00) nurses and 35% (OR 0.39, 95%CI 0.24–0.62) of pharmacists. Hospitals were significantly (83% in teaching hospitals, 79% in regional hospitals, p = < 0.01) more likely to have antibiotic policies, when compared to primary care. A surveillance mechanism for antibiotic consumption was reported in 58% (104/178) of teaching hospitals and 62% (98/159) of regional hospitals. Antimicrobial resistance, patient needs, policy, peer influence and specialty level culture and practices were deemed important determinants for decision-making.

**Conclusion:**

Postgraduate training and support in antibiotic prescribing remains low amongst nurses and pharmacists. Whilst antibiotic policies and committees are established in most institutions, surveillance of antibiotic use is not. The impact of specialty level culture, and peer influence appears to be important factors of antibiotic prescribing.

## Background

The threat of antimicrobial resistance is driving initiatives across the one health care economy [1, 2]. Whilst it is a well-known fact that 80% of antibiotic prescribing for humans occurs in primary care [3] the choice and route of antibiotic agents remains largely limited in that setting. Though antibiotic use in secondary care comprises only 20% of overall human antibiotic consumption, the risks and unintended consequences of antibiotic use in secondary care are greater and more significant than in primary care. Patients in hospitals represent the frail, elderly, very young, and very sick. There is an expectation on all healthcare providers, across the globe, to have put in place appropriate measures to tackle antimicrobial resistance as part of antibiotic stewardship programmes. This expectation however, does not take into consideration the cultural and contextual drivers at national, local and organizational level. Nor does it consider the available resources and active stakeholders in potential stewardship initiatives at the local level.

To address the global challenges of implementing antibiotic stewardship, in 2015, an international group of researchers and clinicians developed a six-week Massive Open Online Course targeting a global audience [4]. The MOOC was designed and developed by an international group of researchers and healthcare professionals with experience in the design and implementation of antibiotic stewardship programmes. The course was first launched in English as an online resource in 2015 (https://www.futurelearn.com/courses/antimicrobial-stewardship). Since then it has been translated to Spanish, Russian and Mandarin (2017) with several runs each year. The MOOC has reached a global audience and the feedback so far suggests it was effective in engaging with participants on the topic of stewardship [4]. There are six modules spread over 6 weeks of learning. Each week has 3 h of learning material which the learners could complete in their own time. Week five of the course is dedicated to ‘Behaviour Change in Antibiotic Prescribing’ and was developed by EC and PD. The content of week five is based on the social science research investigating how contextual knowledge can be used to develop bespoke stewardship interventions in different settings [5]. This is particularly important when considering the variation in resources, both financial and workforce across high, low, and middle income countries and how this affects stewardship [6]. Recognising the international reach of the MOOC, we designed a survey to be distributed amongst the learners of week five this course, during its first run. The survey aimed to investigate what stewardship activities existed in the organisations and countries which were represented by the learners. The survey also explored and what factors influenced the learners’ own antibiotic prescribing decision-making.

## Methods

### Data collection

To investigate the existing stewardship activities, including education and training, in the participants’ organisations and countries and to explore what influenced the learners’ own antibiotic prescribing decision-making. a survey questionnaire (available as supplementary material) was developed using data from previous studies [7], and with input from healthcare professionals in India. In addition to basic demographic questions, the questionnaire asked for details of any existing stewardship programmes in place in the home institution of the participants and any post-graduate training they might have received to support their stewardship activities. Additionally, participants were asked to rank their motivations for antibiotic prescribing. The key questions asked in the survey together with the results are presented in the tables and figures in this paper.

The questionnaire was piloted with healthcare staff in England. All learners participating in week five of the MOOC course were invited to complete the questionnaire online. The survey was placed as an optional activity halfway through the week’s course material. The invitation to participate included a study information sheet and learners were informed that completion of the survey was not mandatory with learners having the option to choose to carry on with the week material without completing the survey (available as supplementary material). All the data from the completed forms were automatically collated and extracted into an excel sheet. The data entry and collection was anonymous and no learner identifier data was collected.

### Statistical analysis

The descriptive data were analysed by institution type and profession. For the statistical analysis, responses of ‘not applicable’ were combined with ‘no’ when analysing the response to whether individuals had received postgraduate training. The categorical variables were analysed using chi-squared test. Cronbach’s alpha test was performed to test the co-efficient alpha for overall reliability of the Likert questions. The Likert questions investigating the perceived determinants of antibiotic prescribing were analysed using an ordinal regression model. All the determinants (including costs, patient needs, colleague recommendations, senior opinion, specialty level culture and practice and policy and guidelines) were fitted into the model to predict their perceived influence on prescribing behaviours. The *p*-values were considered as two-tailed and a p-value of < 0.05 was set as significant. Wherever a *p* value of 0.000 was reported in the software, it was presented as < 0.01 in the results. All analyses were conducted in SPSS 24 (IBM Corp., released 2016), and Microsoft Excel 2013 (Microsoft).

## Results

The first run of the MOOC had 15,570 unique registrations. Of these 6839 individuals from 164 countries completed at least one of the six modules. In total, 920 learners continued to, and completed week five. Of the week five learners, there were 505/920 (55%) survey respondents from 53 countries. The top five countries with the highest frequency of survey responses comprise 65% (327) of the results, summarised in Table 1. The MOOC was, and remains “open source”, meaning any person, including members of the public can complete the course and this was reflected in the survey respondents. Since we were primarily interested in the responses of healthcare professionals (classified as nurses, doctors and pharmacists for the purpose of this study) any respondents outside of this classification were grouped together as other (104/505, 21%), Table 1. Students (27/104), and biomedical scientists and laboratory staff (20/104) were the largest group in the other category, there were also members of the public, dentists, veterinarians, and lecturers, as well as members of the public.

**Table 1:** The survey respondents by profession, the top five countries with most respondents presented in order

Countries	Respondents n	Profession n(%) Doctor	Nurse	Pharmacist	Other
UK	208	60 (29)	55 (26)	40 (19)	53 (25)
Australia	59	10 (17)	7 (12)	38 (64)	4 (7)
India	24	18 (75)	0 (0)	1 (4)	5 (20)
Ireland	21	3 (14)	3 (14)	10 (48)	6 (29)
United States	15	2 (13)	0 (0)	3 (20)	10 (67)
All other countries	178	89 (50)	24 (14)	38 (21)	26 (15)
Total n (%)	505	182 (36)	89 (18)	130 (26)	104 (21)

There were 337/505, (66%) respondents from hospitals, with 178/505 (35%) from a teaching hospital and 159/505 (31%) from a regional hospital (Table 2). Ten percent (49/505) of the respondents were from primary care. Doctors were significantly more likely to have received post-graduate training in infection management and antibiotic stewardship (56%, *p* < 0.01) compared with pharmacists (35%, *p* = 0.44), and nurses (43%, *p* = 0.06) (Table 3). Doctors (141/182, 77%) reported prescribing antibiotics as part of their daily job, with 62% (113/182) reporting reviewing prescriptions of antibiotics. Doctors also reported teaching about infection diagnosis and treatment (113/182, 62%), and developing antibiotic policy and guidelines (85/182, 47%). A small number (19/130, 15% of pharmacists also reported prescribing antibiotics to be part of their daily practice. Amongst the nurses 40% (36/89) reported that they review prescribed antibiotic courses, 43% (38/89) reported teaching on the subject, and 18% (16/89) reported developing stewardship related policy and guidelines, Table 3.

**Table 2:** All respondents by profession and institution

	Profession n (%)	Teaching Hospital	Regional Hospital	Primary Care	Other organisation
Doctor	182 (36)	75 (41)	54 (30)	21 (12)	32 (18)
Pharmacist	130 (26)	41 (31)	55 (42)	11 (8)	23 (18)
Nurse	89 (18)	32 (36)	24 (27)	8 (9)	25 (28)
Other	104 (13)	30 (25)	26 (35)	9 (8)	39 (22)
Total	505 (100)	178 (35)	159 (31)	49 (10)	119 (24)

**Table 3:** Respondent post-graduate education and training and roles in antibiotic management by profession

	Profession n	Received Post graduate training n (%)			As part of your job do you do any of the following in relation to antibiotics? N(%)				
		Yes	No	Odds Ratio (95% CI)	Prescribe	Administer	Review	Teach about infection diagnosis/treatment	Develop antibiotic policy/guidelines
Doctor	182	105 (58)	77 (42)	Ref 0.39 (0.24–0.62) 0.59 (0.35–1.00)	141 (77)	35 (19)	112 (62)	113 (62)	85 (47)
Pharmacist	130	45 (35)	85 (65)		19 (15)	10 (8)	117 (90)	58 (45)	74 (57)
Nurse	89	38 (43)	51 (57)		14 (16)	42 (47)	36 (40)	38 (43)	16 (18)

Hospitals (teaching and regional) were significantly more likely to have an institutional antibiotic related policy or guidelines when compared to primary care (p = < 0.01), Table 4. Hospitals were more likely to have a committee designated to stewardship (*p* < 0.01) (teaching hospitals 73% 130/178, 77% 123/159, regional hospitals) when compared to primary care (33% 16/49). A surveillance mechanism for antibiotic consumption and prescribing was present in 58% (104/178) of teaching hospitals and 62% (98/159) of regional hospitals.

**Table 4:** Antibiotic stewardship activities by institution

Institution	n	Do you have antibiotic policy or guideline in your organistion?			Are there specific committees/groups dedicated to AMS?			Is there a reporting structure for antibiotic use and prescribing?		
		Yes n(%)	No n(%)	*P* Value	Yes n(%)	No n(%)	*P* Value	Yes n(%)	No n(%)	*P* Value
Teaching Hospital	178	147 (83)	31 (17)	< 0.01*	130 (73)	48 (27)	< 0.01*	104 (58)	74(42)	< 0.01*
Regional Hospital	159	126 (79)	33 (21)		123 (77)	36 (23)		98 (62)	61(38)	
Primary Care	49	32 (65)	17 (35)		16 (33)	33 (67)		21 (43)	28 (57)	
Other	119	62 (52)	57 (48)		57 (48)	62 (52)		47 (39)	72 (61)	
Total	505	367 (73)	138 (27)		326 (65)	179 (35)		270 (53)	235 (47)	

^*^Statistical significance calculated using Pearson Chi Squared test

**Fig. 1:**
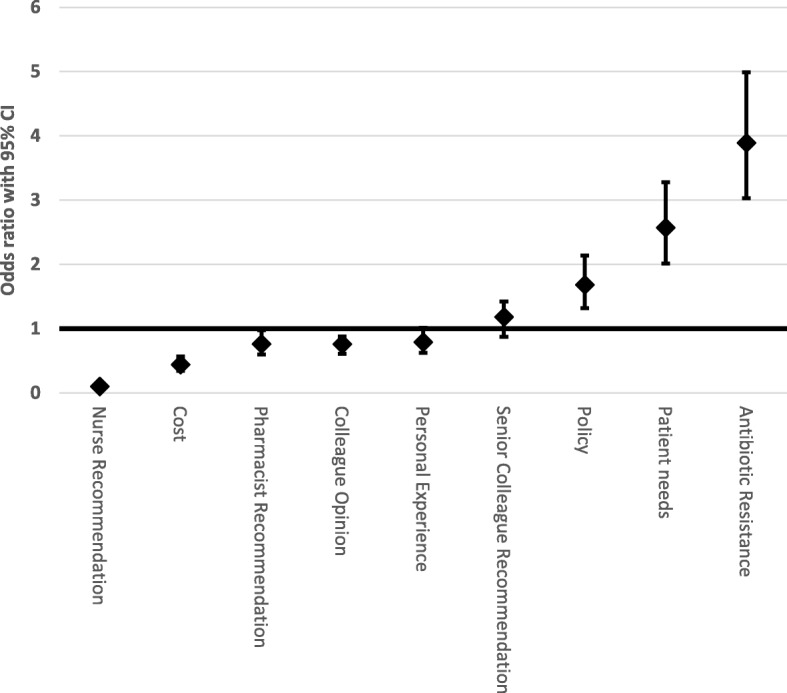
Figure presenting the trend in the odds ratios of respondents self-perceived determinants of antibiotic prescribing, relative to culture and practice at specialty level (set as 1)

The pattern of scoring to the Likert questions on perceived influences on antibiotic decision making were similar across the professions. The highest mean scores on the Likert scale were for antimicrobial resistance, policy, patient needs and senior colleague recommendations as respondent perceived determinants on antibiotic prescribing. When looking at the odds ratios of the mean rankings for each variable reported to influence decision making, antimicrobial resistance, patient needs, policy, and specialty level culture and practices were deemed the most influential factors in this cohort (Fig. 1). Nurse recommendation, and cost were the least influential factors. When compared with the odds ratio for specialty level culture and practices, antibiotic resistance, patient needs, and policy were all significantly more influential in decision making (*p* < 0.01). The opinion of colleagues, pharmacist recommendation, and senior doctor recommendation were ranked very closely in influence on specialty level and practices (Fig. 1). To assess the internal consistency of the Likert questions, a Cronbach’s alpha test was performed, and a value of 0.744 was reported. This demonstrates a good co-efficient alpha for overall reliability of the Likert questions.

## Discussion

This survey had a high (55%) response rate from the participants of week five of the MOOC. Most of the survey respondents, however were from the UK and Australia, and five countries comprised 65% of the respondents. This may have been due to the MOOC being in English, and therefore not accessible to non-English speakers, although, responses from the USA and Canada with English speaking populations, and with established stewardship programmes in their healthcare system were low. It was encouraging to see participation from nurses and pharmacists, in addition to doctors, in this survey. Though this course was targeted healthcare professionals in hospitals, it attracted learners from primary care as well as scientists, students and from other wider members of the population. This interest from the non-healthcare professional workforce in an activity about antibiotic stewardship is a positive step in raising awareness about the need to engage with the wider population on the necessity to conserve antibiotics.

The participation of nurses and pharmacists in stewardship activities such as developing policy and guidelines, and reviewing antibiotic prescriptions is a positive step to a broader inclusion of healthcare professionals, globally in stewardship efforts. Although pharmacist participation in stewardship activities is the norm in some healthcare systems such as in USA, UK, Canada and Australia, in many other parts of the world, stewardship remains the responsibility of doctors [8]. The reported nurse involvement and participation is encouraging the see, though it has not been reported in other surveys and studies. Involving the wider healthcare workforce in stewardship is a key gap that needs to be addressed [9, 10]. Whilst culturally, and historically, the expectation is that doctors lead decision making in the clinical setting, the participants in this survey, representing 53 countries, demonstrate that other healthcare professionals can and do have a role to play in stewardship.

The most common components of stewardship are reported to be the use of policy and guidelines, the review of antibiotic prescriptions, and the existence of dedicated committees to stewardship. The availability and reporting of antibiotic consumption and surveillance reports however, remain low at 58% in teaching hospitals, and 62% in regional hospitals, and 43% in primary care. In general, stewardship activities were significantly more likely to be present in hospitals than in primary care. Since this is a survey with responses from 53 countries, the results will reflect the heterogeneity of the participants’ national healthcare systems. Education and training in infection management and antibiotic stewardship, at the postgraduate level, remains a resource primarily available to doctors. This may be one of the reasons why it is more difficult for other healthcare professionals to become involved and participate in stewardship activities. Education and training in this field is not universal and requires further resources, particularly in healthcare settings where traditionally doctors are still considered to be the main decision makers and leaders in infection management and antibiotic stewardship.

When asked to rate the factors that influence antibiotic decision making, all three professions were consistent in their rankings of the key factors, namely: antimicrobial resistance, patient need, local policies. These determinants were closely followed by the influence of senior doctors, local culture and practices and personal experience as key determinants of antibiotic decision making. This finding of the influence of senior doctors and specialty level and local culture is similar to findings from in-depth qualitative studies investigating the determinants of antibiotic decision-making [7, 11]. To develop sustainable and effective interventions in antibiotic stewardship it is important to recognise and address these cultural determinants to shape the outcome of stewardship interventions. The general MOOC course participants were invited to respond to a post-course survey, to which 219/6839 participants responded. The findings of this survey have been published, and report 95% (208/219) of learners rating the course as good or excellent, with week five of the course scoring most favourably with learners [4]. Furthermore, the discussion forum for week five of the MOOC provided ample feedback on the relevance and importance of understanding the social and behavioural aspects of antibiotic prescribing. This feedback is available in the week five course content (https://www.futurelearn.com/courses/antimicrobial-stewardship).

### Limitations

This survey was conducted across a group of individuals who had registered to participate in a course on antibiotic stewardship. There will have been inherent selection bias in this group of respondents. The data is from 2015, and since then there has been further progress in supporting antibiotic stewardship programmes both internationally and nationally.

## Conclusions

This study with participants representing 53 countries provides insights into the main components of antibiotic stewardship programmes in different countries and the key factors which influence the antibiotic decision-making of key healthcare professionals. Whilst antibiotic policies and committees are established in most institutions, surveillance of antibiotic use is not universal. Postgraduate training and support in antibiotic prescribing remains low amongst nurses and pharmacists. The impact of specialty level culture, and peer influence appears to be significant factors of antibiotic decision-making. These data identify existing gaps in adequate training and education for staff involved in stewardship, and the need for greater participation of nurses and pharmacists to ensure effective and sustainable stewardship programmes.

## Abbreviations

MOOC: Massive Open Online Course
